# Adjuvant chemotherapeutic treatment of 1650 patients with early breast cancer in routine care in Germany: data from the prospective TMK cohort study

**DOI:** 10.1007/s12282-017-0823-7

**Published:** 2017-12-04

**Authors:** Steffen Dörfel, Claus-Christoph Steffens, Dirk Meyer, Hans Tesch, Lisa Kruggel, Melanie Frank, Martina Jänicke, Norbert Marschner, The TMK-Group (Tumour Registry Breast Cancer)

**Affiliations:** 1 OncoCentre Dresden/Freiberg, Leipziger Str. 118, 01127 Dresden, Germany; 2 Centre for Haematology and Oncology, Harsefelder Str. 6-8, 21680 Stade, Germany; 3 Centre for Oncology, Nikolausberger Weg 36, 37073 Göttingen, Germany; 4 Centre for Haematology and Medical Oncology at Bethanien, Im Prüfling 17-19, 60389 Frankfurt/Main, Germany; 5 Clinical Epidemiology and Health Economics, iOMEDICO, Hanferstr. 28, 79108 Freiburg, Germany; 6 Statistics, iOMEDICO, Hanferstr. 28, 79108 Freiburg, Germany; 7 Outpatient-Centre for Interdisciplinary Oncology and Haematology, Wirthstrasse 11c, 79110 Freiburg, Germany

**Keywords:** Breast neoplasms, Registries, Cohort studies, Taxoids, Chemotherapy, Adjuvant

## Abstract

**Background:**

Several regimens for which efficacy was established in randomized controlled trials are recommended in current treatment guidelines for early breast cancer. However, knowledge on use and effectiveness of commonly administered chemotherapeutic agents in real-life care and across all breast cancer subtypes is limited.

**Methods:**

The prospective, multicentre German TMK cohort study (Tumour Registry Breast Cancer) recruited patients in 148 oncology outpatient-centres. Data from 1650 patients who completed adjuvant chemotherapy were analysed regarding treatment regimens and taxane use from 2007 to 2014. The association of patient characteristics with application of taxane-free regimens was examined with a multivariate regression model.

**Results:**

The preferred adjuvant treatment shifted from fluorouracil, anthracycline and cyclophosphamide containing regimens to anthracycline/taxane combinations. Taxane use increased for all subtypes, and the greatest rise was among node-negative patients. Older age, node-negativity, lower grading, HR-positive/HER2-negative subtype and earlier start year of therapy were significantly associated with taxane-free therapy.

**Conclusions:**

Treatment with anthracycline/taxane-based chemotherapy in Germany has been rising for every subtype. The increased taxane use reflects updated guideline recommendations over the past decade. Cohort studies like the TMK provide insight into real-life treatment of patients outside of clinical trials.

**Electronic supplementary material:**

The online version of this article (10.1007/s12282-017-0823-7) contains supplementary material, which is available to authorized users.

## Introduction

Breast cancer (ICD-10 C.50), with an annual incidence of 70,000 new cases, is the most common type of cancer among women in Germany, and the second most frequent cause of cancer-related death in women [1]. Implementation of screening procedures and development of new therapies revealed constant mortality rates despite increasing incidence; age-standardised mortality declined slightly and the 5-year relative survival increased over the past decade [2, 3].

Standard therapy of patients with early breast cancer consists of surgery, radiation and adjuvant systemic therapy. However, as breast cancer is highly heterogeneous, the selection of adjuvant systemic therapy depends on stage, histology and on molecular subtypes of the tumour [4, 5]. Current adjuvant systemic therapy options include chemotherapy, endocrine therapy for hormone receptor (HR)-positive tumours, and targeted biological agents such as trastuzumab for human epidermal growth factor receptor (HER2)-positive tumours. The treatment decision is based on multiple factors and includes—in addition to tumour biology and the predicted sensitivity to particular treatment types—the patients’ physical constitution, biological age and comorbidities as well as patients’ preferences.

The current St. Gallen international experts consensus recommends endocrine therapy alone for adjuvant systemic therapy of luminal A-like breast cancer subtypes with low risk in the majority of cases, while additional chemotherapy should be considered in patients with four or more lymph nodes involved [6]. For luminal B-like HER2-negative subtypes, endocrine therapy and chemotherapy is recommended in the majority of cases, while for luminal B-like HER2-positive subtypes, chemotherapy, anti-HER2 targeted therapy and endocrine therapy are recommended for all patients. For the triple-negative subtype [oestrogen-receptor (ER)-negative, progesterone receptor (PR)-negative and HER2-negative], chemotherapy should include an anthracycline and a taxane [6].

Despite these recommendations and other clinical practice guidelines, the extent to which these recommendations are incorporated into routine clinical practice is only partially known [7–9] and often limited by retrospective data collection [10].

There are several regimens for which treatment efficacy has been established in randomized controlled trials (RCTs) [11]. These regimens differ in duration, the combination and the dosages of drugs given. Research in clinical trials usually focuses on treatment with one regimen or drug of interest and also applies stringent selection criteria. As more treatment options have become available, a wider variety of treatments was applied to individual patients in everyday routine care. However, knowledge on the use of existing chemotherapeutic agents and combinations in routine care is limited. Clinical cohort studies like ours can help to fill this gap and provide insight into treatment and outcome of patients in routine care [12].

Today, most systemic breast cancer treatments can be delivered on an outpatient basis. In Germany, ambulatory care is predominantly provided by office-based specialists and hospital outpatient centres. This paper focuses on the cytotoxic treatment of patients with early breast cancer in daily routine practice as well as changes in treatment over time from 2007 to 2014.

## Patients and methods

### Data source

The Tumour Registry Breast Cancer (TMK) is an ongoing, open, prospective, longitudinal, observational, multicentre study for patients with breast cancer. The TMK was established by office-based medical oncologists. The study was approved by the responsible ethics committee and is registered at ClinicalTrial.gov (NCT01351584). Recruitment started in February 2007. 2250 patients with curative treatment intention were enrolled until April 2014, and 2250 patients with palliative treatment intention have been recruited until May 2016. 148 outpatient centres and clinics for medical oncology located all over Germany are actively participating in the TMK. 122 such sites enrolled patients with adjuvant treatment intention. The sites were encouraged to enrol patients consecutively to ensure unselected recruitment. Eligible patients are women aged ≥ 18 years with histologically confirmed breast cancer and systemic antineoplastic treatment. Written informed consent was obtained from all patients. A maximum of 6 weeks time difference was allowed between start of systemic therapy and signed informed consent. The TMK has previously been described in detail [12].

At enrolment, data on all previous cancer treatments, patients’ socio-demographics and tumour characteristics (tumour location, histology, stage, grading, ER-, PR- and HER2-receptor status) are documented. Comorbidity is assessed using the updated Charlson Comorbidity Index (CCI) [13]. Patients are treated according to physicians’ choice and visits are scheduled according to their individual treatment regimen. No specifications are imposed on the physicians’ assessment for treatment at any time. All patients are followed for up to 5 years from enrolment or until death, loss to follow-up or withdrawal of consent. During the follow-up period, data on all systemic antineoplastic treatments, radiotherapies and surgeries, diagnostic follow-up controls as well as on outcome and course of the disease are collected. Systemic therapies are documented by listing all agents separately and not as predefined regimens to allow for documentation of individual combinations. Patient data are transferred from medical records to a secure web-based electronic case report form (eCRF) by designated site staff and are updated after each follow-up examination, any change in therapy or at least every 6 months. For quality assurance, automated data plausibility checks are performed and queries are generated by the eCRF software. Manual checks on data completeness and plausibility as well as spot site monitoring are performed regularly to ensure reliability.

### Cohort definition

**Fig. 1:**
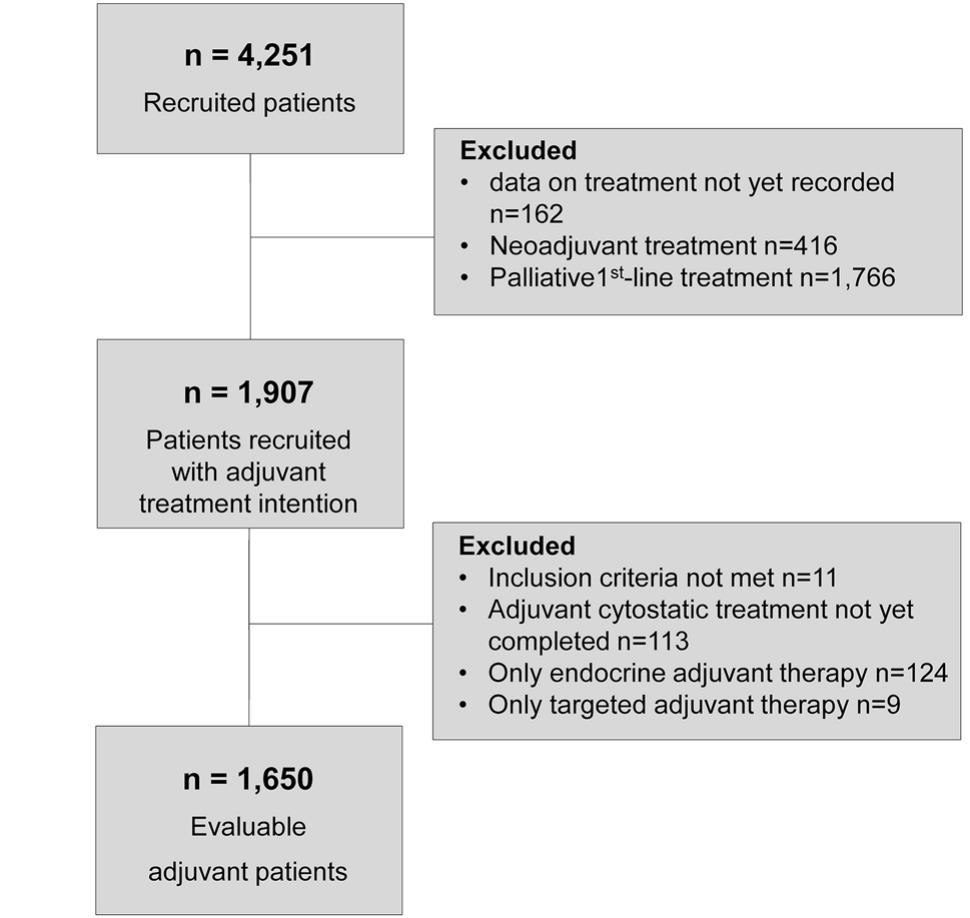
Cohort definition. Number of patients enrolled in the TMK until October 2014

Data cut-off for the present analysis was October 2014. By then, 4251 patients had been recruited into the TMK. Of these, 1907 patients were recruited at start of systemic adjuvant treatment. The present analysis is based on 1650 patients who completed adjuvant chemotherapy. Details of the patient flow are presented in Fig. 1.

### Statistical analysis

The combinations of documented chemotherapeutic agents were coded into chemotherapy regimen. Anthracycline-based chemotherapy was defined as receiving epirubicin or doxorubicin (A) alone or in combination with any other antineoplastic drug except paclitaxel or docetaxel. Taxane-based chemotherapy was defined as the use of paclitaxel (P) or docetaxel (D) alone or in combination with any other antineoplastic drug except epirubicin or doxorubicin. Combination therapy with anthracycline and taxane was defined as receiving epirubicin or doxorubicin and paclitaxel or docetaxel, with or without any other additional antineoplastic drug. HR-status was assessed as combination of ER- and/or PR-positive (HR-positive) or both receptors negative (HR-negative). Descriptive analyses were performed using SPSS v.20.0 (IBM Corp.).

Multivariate logistic regression analysis was used to examine the association of patient and tumour characteristics and treatment with taxane-containing chemotherapy (yes/no) as dependent variable. Potential baseline variables for the multivariate regression model were as follows: age at start of therapy (in 10 years increments), CCI, the institution deciding on adjuvant therapy, tumour size, nodal status, grading, molecular subtype and year of treatment start. Based on a stepwise backward selection of variables, using the likelihood ratio test for model comparison, CCI and the institution deciding on adjuvant therapy were not included in the final model. Analysis was performed using R version 2.15.1. The results were displayed as odds ratios (OR) with 95% confidence intervals (95% CI) and *p* values. The significance level was set at 0.05.

## Results

### Patient and tumour characteristics

Table 1 presents demographic and clinical characteristics of patients with early breast cancer at the start of adjuvant chemotherapy. Characteristics are shown for the entire cohort and for patients treated with one of the four most frequent regimens (received by at least 10% of patients). A table listing the characteristics for all regimens is available in the supplemental material (Table S1). Median age at start of therapy was 56.7 years, 13% of the patients were aged ≥ 70. HR- and HER2-status were documented for 98% (*n* = 1611) of the patients. More than half (59%) of the patients had HR-positive/HER2-negative tumours, 25% HER2-positive tumours and 16% triple negative tumours. 47% of patients were node-negative. 26% of the patients were pre-menopausal and 58% were post-menopausal. 53% of the patients had at least one comorbidity, with hypertension (28%) and diabetes (8%) recorded most frequently. 24% of the patients were obese (BMI > 30). The majority of patients underwent breast conserving surgery (BCS 71%), 26% underwent mastectomy. 86% of the patients with BCS received radiotherapy post-surgery, in contrast to 60% of the patients who underwent mastectomy. For more details, see supplemental Table S1.

**Table 1:** Patient characteristics at time of enrolment, split up according to the most common chemotherapy regimen

	F + A + C (*n* = 456)	F + A + C + D (*n* = 413)	A + C + P (*n* = 279)	A + C + D (*n* = 178)	All patients (*N* = 1650)
Age at start of therapy (*n*)*	456	413	278	178	1649
Median (years)	56.0	56.6	56.7	53.3	56.7
BMI (*n*)*	453	404	275	176	1626
Mean (kg/m^2^) ± SD (kg/m^2^)	26.8 ± 5.3	26.8 ± 5.3	26.5 ± 5.2	26.4 ± 4.9	26.8 ± 5.2

	*n*	%	*n*	%	*n*	%	*n*	%	*n*	%
Any comorbidity^a^	235	51.5	192	46.5	153	54.8	91	51.1	876	53.1
CCI = 0	394	86.4	376	91.0	245	87.8	165	92.7	1443	87.5
CCI = 1	29	6.4	13	3.1	15	5.4	4	2.2	71	4.3
CCI ≥ 2	33	7.2	24	5.8	19	6.8	9	5	136	8.2
Hypertension	132	28.9	101	24.5	79	28.3	34	19.1	458	27.8
Diabetes mellitus	35	7.7	24	5.8	21	7.5	9	5.1	128	7.8
Cardiovascular disorders	6	1.3	3	0.7	5	1.8	1	0.6	39	2.4
Tumour subtype (*n*)^a^	442		403		275		175		1611	
HR-positive/HER2-negative	301	68.1	254	63.0	165	60.0	104	59.4	949	58.9
HER2-positive	83	18.8	87	21.6	61	22.2	29	16.6	402	25.0
Triple negative	58	13.1	62	15.4	49	17.8	42	24.0	260	16.1
Tumour stage^a,b^	403		364		248		162		1456	
I	197	48.9	59	16.2	33	13.3	31	19.1	407	28.0
II	194	48.1	245	67.3	115	46.4	80	49.4	763	52.4
III	12	3.0	60	16.5	100	40.3	51	31.5	286	19.6
Nodal stage^a^	456		413		279		178		1650	
N−	376	82.5	119	28.8	81	29.0	58	32.6	781	47.3
N+	77	16.9	291	70.5	195	69.9	117	65.7	853	51.7
NX	3	0.7	3	0.7	3	1.1	3	1.7	16	1.0
Local therapy^a^	456		413		279		178		1650	
BCS	362	79.4	296	71.7	173	62.0	120	67.4	1166	70.7
Post-BCS radiotherapy^c^	322	89.0	258	87.2	138	79.8	109	90.8	1001	85.8
Mastectomy	87	19.1	105	25.4	92	33.0	50	28.1	435	26.4
Post-mastectomy radiotherapy^d^	28	32.2	74	70.5	65	70.7	33	66.0	259	59.5
Surgery unknown	7	1.5	12	2.9	14	5.0	8	4.5	49	3.0

Only the four mainly used regimens are shown; each regimen could be administered with or without additional HER2-inhibitor trastuzumab and/or additional endocrine therapy

*A* epirubicin or doxorubicin, *BCS* breast conserving surgery, *BMI* body mass index, *C* cyclophosphamide, *CCI* Charlson comorbidity index, *D* docetaxel, *Car* carboplatin, *F* fluorouracil, *P* paclitaxel, *SD* standard deviation

^a^Number of patients with data available on the respective parameter at time of enrolment

^b^Tumour stage according to American Joint Committee on Cancer. 7th ed. New York, NY: Springer; 2010

^c^Percentages refer to all patients who received BCS

^d^Percentages refer to all patients who received mastectomy

### Chemotherapy regimen

**Fig. 2:**
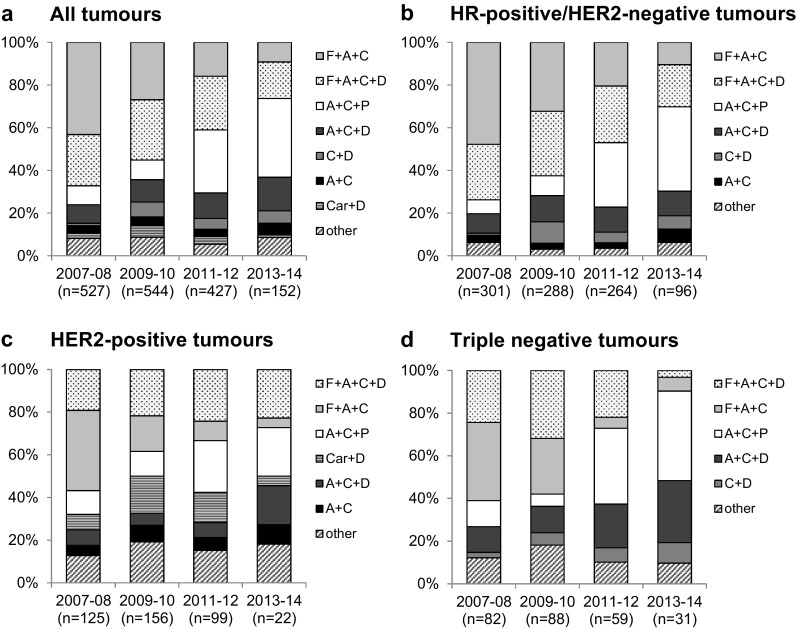
Frequency of most commonly used regimens over time. Shown are the regimens used in more than 5% of the patients for **a** all patients, **b** patients with HR-positive/HER2-negative tumours, **c** patients with HER2-positive tumours and **d** patients with triple negative tumours. Each regimen could be administered with or without additional HER2-inhibitor and followed by additional endocrine therapy. *A* epirubicin or doxorubicin, *C* cyclophosphamide, *D* docetaxel, *Car* carboplatin, *F* fluorouracil, *P* paclitaxel

Figure 2a shows the most frequently administered chemotherapy regimens over time for all patients. Most regimens were based on the combination of fluorouracil (F), epirubicin or doxorubicin (A) and cyclophosphamide (C). F + A + C was given to 27% of the patients (*n* = 456), F + A + C in combination with docetaxel (D) to 25% of the patients (*n* = 413). 17% of the patients (*n* = 279) received A + C in combination with paclitaxel (P), 11% A + C in combination with docetaxel (*n* = 178). Less frequently used were the taxane-based regimens C + D and carboplatin + D (5 and 4%, respectively) and the anthracycline based, taxane-free regimen A + C (4%). Over time, a shift from the fluorouracil- and anthracycline-based regimen F + A + C ± D to anthracycline and taxane combinations (A + C + D and A + C + P) can be observed for all patients, as well as within the subgroups with HR-positive/HER2-negative, HER2-positive and triple negative tumours (Fig. 2b–d). For 81% of the patients with HER2-positive tumours, an additional trastuzumab therapy was documented (corresponding to 89% of the premenopausal and 80% of the postmenopausal patients, respectively). 82% of the premenopausal and 81% of postmenopausal patients with HR-positive tumours received endocrine therapy after cytotoxic (and possible HER2-targeted) therapy.

### Chemotherapy with and without taxanes

**Fig. 3:**
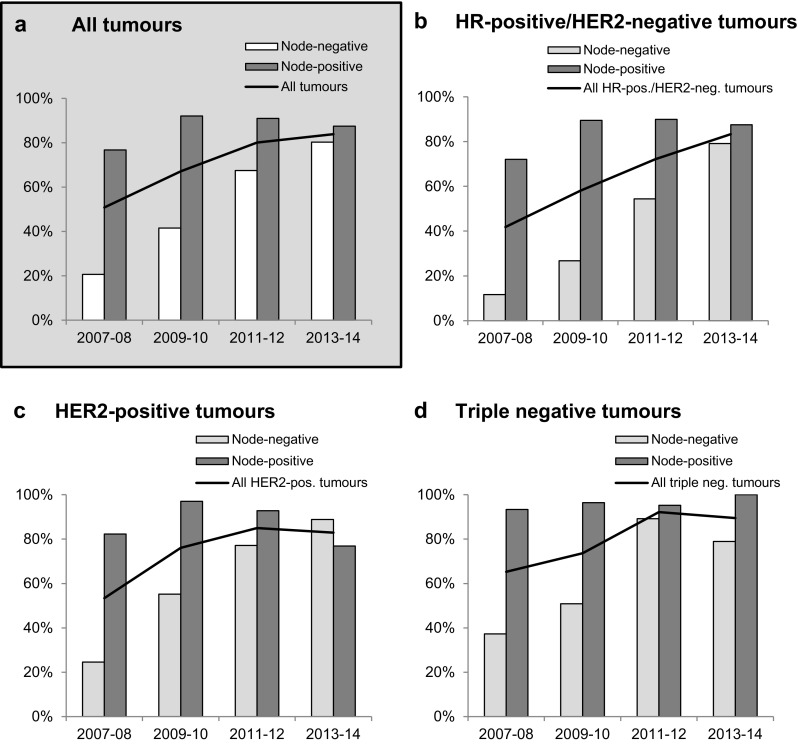
Frequency of taxane-use over time. **a** All patients as well as split up according to the subgroups, **b** HR-positive/HER2-negative, **c** HER2-positive and **d** triple negative. The line depicts the average for each group over time. *pos.* positive, *neg.* negative

Looking at all patients and all chemotherapy treatments (including those not shown in Table 1), 32% of the patients (*n* = 528) received anthracycline-based chemotherapy without a taxane, 10% (*n* = 163) received taxane-based chemotherapy without an anthracycline, and 57% (*n* = 938) received an anthracycline/taxane-combination, with an increase over time from 46% in 2007–2008 to 75% in 2013–2014 (Fig. 3a). Only 1% of the patients (*n* = 21) received chemotherapy without anthracyclines or taxanes. The constant increase of patients treated with taxane-based regimen within all subgroups is displayed in Fig. 3. However, when stratified by nodal stage, this increase seems to be a result of the rising proportion of node-negative patients receiving taxanes (from 21% in 2007–2008 to 80% in 2013–2014, Fig. 3a), while in node-positive patients the use of taxane-based regimens was already very common at the start of the observation period, with a slight further increase (77% in 2007–2008 and 88% in 2013–2014, Fig. 3a). This pattern is present within all subgroups analysed (Fig. 3b–d). The largest increase could be observed for node-negative patients with HR-positive/HER2-negative tumours. Here, taxane use increased from 12% in 2007–2008 to 79% in 2013–2014 (Fig. 3b). Table 2 presents the association of baseline patient and tumour characteristics of patients treated with a taxane-containing regimen. HER2-positive or triple negative tumour subtype, bigger tumour size, node-positivity, higher tumour grading and a more recent start year of treatment were significantly associated with receiving taxane-based regimens.

**Table 2:** Multivariate analysis for the odds of chemotherapy with taxanes

Parameter	Multivariate analysis		
	OR	95% CI	*p* value
Age at start of therapy (10 years)	0.84	0.75–0.95	0.0039**
Tumour subtype			
HER2-pos. vs. HR-pos./HER2-neg.	2.29	1.66–3.15	< 0.0001***
Triple neg. vs. HR-pos./HER2-neg.	3.03	2.04–4.51	< 0.0001***
Tumour size^a^			
> T1 vs. T1	1.42	1.10–1.84	0.0073**
Tis vs. T1	2.97	0.91–9.62	0.0703
TX vs. T1	4.40	0.28–68.03	0.2894
Nodal stage^a^			
N+ vs. N−	15.0	11.02–20.41	< 0.0001***
NX vs. N−	2.34	0.71–7.73	0.1650
Tumour grading^a^			
G2 vs. G1	1.92	1.12–3.29	0.0171*
G3 vs. G1	2.71	1.55–4.75	0.0005***
GX vs. G1	3.65	0.89–14.98	0.0718
Start of treatment (years)	1.56	1.45–1.68	< 0.0001***

Intercept OR 0.11, CI 0.05–0.26, *p* value < 0.0001

40 patients were excluded from this analysis due to missing covariates. CCI and the institution deciding on adjuvant therapy were not included in the final model

*CI* confidence interval, *neg.* negative, *OR* odds ratio, *pos.* positive

*p < 0.05, **p < 0.01, ***p < 0.001

^a^According to American Joint Committee on Cancer, 7th edn. New York, NY: Springer; 2010

In contrast, older patients were less likely to receive taxane-based therapy. Node-positive patients were 15 times more likely to receive taxane-based regimens than node-negative patients. Patients with triple negative tumours were 3 times more likely to receive taxane-based regimens than patients with HR-positive/HER2-negative tumours. Furthermore, patients with poorly differentiated tumours (G3) were 2.7 times more likely to receive taxane-based regimens compared to patients with well-differentiated (G1) tumours. The start year of therapy was associated with 1.56 increased odds of receiving taxane-based regimens for every year later than 2007.

## Discussion

The aim of this analysis was to describe the different adjuvant cytotoxic treatment approaches for patients with early breast cancer in daily routine practice in Germany as well as to identify changes in treatment strategies since 2007. The strengths of our study are the use of prospectively collected clinical data on systemic therapy in unselected primary breast cancer patients throughout Germany. The TMK is not limited to patients treated with a particular substance, thus providing a unique assessment of the different systemic treatment strategies applied outside of clinical trials, reflecting the “real-world” setting. Furthermore, we show a comprehensive analysis of treatment patterns of the breast cancer subtypes. Outcome data from the TMK will be analysed after an adequate follow-up time is reached, addressing the key question as to how the clinical efficacy shown in RCTs translates into clinical effectiveness in daily routine practice.

Our data show a shift from F + A-based to anthracycline/taxane-based regimens as preferred adjuvant treatment. While the increase of taxane use was apparent in all patient subgroups, the greatest increase was among node-negative patients. Our regression model examined the association of patient and tumour characteristics with receipt of taxane-free adjuvant chemotherapy. Positive nodal stage, triple negative or HER2-positive tumours, tumour grading, year of therapy and tumour size were significantly associated with decreasing odds for taxane-free therapy. Older age was the only factor associated with increasing odds for taxane-free therapy.

In our cohort study, 81% of the patients with HR-positive tumours were treated with endocrine therapy and additional HER2-inhibitors were documented for 81% of the patients with HER2-positive tumours. Our cohort did not include patients treated exclusively with endocrine therapy. In addition, although HR- and HER2-status were documented for 98% of our patients, we cannot distinguish between luminal A and luminal B subtypes because data on Ki-67 status were not collected prior to 2011. Both limitations have to be taken into account when comparing our data with other published studies.

Compared to the patients from different European and Californian registry cohorts, our patients are similar with regard to the proportion of HR- and HER2-receptor subtypes [14–17], as well as distribution of age and CCI [17–20], if known restrictions of these registries (e.g. an age limit of 75) and our primary focus on patients receiving cytotoxic treatment are accounted for. The proportion of patients with CCI ≥ 1 (12%) is also similar to patients in other real-life settings [17–19]. When compared to patients in RCTs, those treated in routine care are considerably different with regard to demographic and clinical characteristics [21]. In our TMK cohort, median age at start of adjuvant systemic treatment is 56 years, compared to approximately 50 years in RCTs [22–24]. Furthermore, the TMK includes patients who would have been excluded from RCTs because of the severity of comorbidities [22–24]. This indicates that the general state of health of our patients is less favourable, compared to patients in prospective clinical trials.

Positive nodal stage is a known predictor of relapse [25, 26] and pivotal studies [27, 28] and meta-analyses [29, 30] have shown a survival benefit of about 3% (5-year survival-rate) by adding a taxane to an anthracycline-based chemotherapy for node-positive patients. Therefore, as expected, the highest rate of combined anthracycline/taxane regimen in our cohort is seen in the node-positive patient population. Nevertheless, the highest increase in taxane therapy can be seen in node-negative patients across all subtypes. The increase in taxane-based chemotherapy since 2008 over all patient subgroups in our cohort reflects changes in the treatment guidelines: while taxanes were not recommended for node-negative patients in 2008–2009, the use of taxanes for all patients receiving chemotherapy has been highly recommended since 2012 by the German AGO-guidelines [31]. However, the benefit of taxane-based chemotherapy for all node-negative patients has yet to be proven. Trials including node-negative patients that showed a survival benefit, either only included high risk patients [23] or showed only a significant benefit for the node-positive subgroup [25]. Node-negative high-risk patients are probably the reason why the EBCTCG meta analysis in 2012 [32] also reported a small but significant reduction of the relative recurrence risk. This means, that some node-negative patients might benefit from taxane-based therapies, especially if other risk factors are present [23]. However, there is also evidence that as many as 70% of node-negative patients could be treated effectively with surgery, radiotherapy and endocrine therapy alone [33, 34]. On the other hand, due to the cardiotoxic side effects of anthracyclines, adjuvant combinations of docetaxel and cyclophosphamide (TC) are recommended for node-negative or low-risk node-positive breast cancer as an alternative to anthracycline-based therapies [35]. Nevertheless, only 10% of the patients of our cohort received anthracycline-free regimens.

Currently it is not possible to reliably identify node-negative patients, who will benefit from anthracycline/taxane-combination therapies. This is probably the main reason why an increasing proportion of node-negative patients in the TMK received such a combination therapy. However, if the overall risk of recurrence is small, the treatment choice has to be counterbalanced with treatment-related acute and long-term toxicities [36–38]. Especially in the light of recent findings regarding treatment choice based on gene-expression profiles, the decision for adjuvant chemotherapy treatment should not be taken lightly: the PlanB trial showed that patients with early HR-positive breast cancer and enhanced risk (assessed by the 21-gene recurrence score) had excellent 3-year survival rates despite omitted chemotherapy [39]. Similarly, 5-year survival rates of patients with high clinical, yet low genomic risk (assessed with the 70-gene signature test), were comparable regardless of the receipt of chemotherapy [40]. On the other hand, it has been shown that the patients’ perceived estimation of increased risk of relapse is a major determinant for using adjuvant chemotherapy despite uncertainties regarding the degree of benefit when added to endocrine therapy in the low-risk HR-positive population. Thus the rise of taxane-use in node-negative patients in the TMK might also be partially attributed to patients’ personal preference.

## Conclusion

In summary, we show that adjuvant treatment with anthracycline/taxane-based chemotherapy has continuously increased in Germany since 2007; with the highest increase seen in the group of node-negative patients. Data from cohort studies like the TMK provide substantial information about the treatment of patients outside of clinical trials, which will help to gain more insight into benefit of changes in treatment strategies and the outcome of patients in routine practice.

## Electronic supplementary material

Below is the link to the electronic supplementary material.

Supplementary material 1 (DOCX 48 kb)
