# Supplementary material for: Adjuvant chemotherapeutic treatment of 1650 patients with early breast cancer in routine care in Germany: data from the prospective TMK cohort study
Source: Breast Cancer. 2017 Dec 4;25(3):275–83. doi: 10.1007/s12282-017-0823-7 (PMC5906523; doi:10.1007/s12282-017-0823-7)
Supplement: Supplementary file 1 — Supplementary material 1 (DOCX 48 kb) [file 12282_2017_823_MOESM1_ESM.docx]

**Table S1. Patient characteristics at time of enrolment, split up according to the 9 most common chemotherapy regimen**

|  | **F+A+C**  **n=456** | | **F+A+C+D n=413** | | **A+C+P**  **n=279** | | **A+C+D**  **n=178** | | **C+D**  **n=75** | | **Car+D**  **n=61** | | **A+C**  **n=62** | | **F+A+C+P n=19** | | **CMF**  **n=17** | | **Total**  **N=1,650** | |
| --- | --- | --- | --- | --- | --- | --- | --- | --- | --- | --- | --- | --- | --- | --- | --- | --- | --- | --- | --- | --- |
| Age at diagnosis [n] * | 456 | | 413 | | 277 | | 178 | | 75 | | 61 | | 61 | | 19 | | 17 | | 1,646 | |
| Median [yrs] | 55.9 | | 56.3 | | 56.3 | | 53.2 | | 65.0 | | 56.6 | | 63.2 | | 60.3 | | 68.4 | | 56.6 | |
| Mean [yrs] ±SD [yrs] | 55.4 | ±10.5 | 56.4 | ±10.1 | 56.2 | ±10.9 | 53.4 | ±10.3 | 62.1 | ±12.4 | 56.6 | ±12.4 | 58.8 | ±18.0 | 58.2 | ±9.6 | 66.2 | ±12.4 | 56.4 | ±11.0 |
| Age at start of therapy [n] * | 456 | | 413 | | 278 | | 178 | | 75 | | 61 | | 62 | | 19 | | 17 | | 1649 | |
| Median [yrs] | 56.0 | | 56.6 | | 56.7 | | 53.3 | | 65.1 | | 56.7 | | 62.9 | | 60.4 | | 68.5 | | 56.7 | |
| Mean [yrs] ± SD [yrs] | 55.6 | ±10.5 | 56.5 | ±10.1 | 56.4 | ±10.8 | 53.6 | ±10.3 | 62.4 | ±12.2 | 57.1 | ±12.0 | 60.5 | ±12.3 | 58.4 | ±9.6 | 66.6 | ±12.6 | 56.6 | ±11.0 |
| > 70 yrs [n, %] | 36 | 7.9 | 45 | 10.9 | 37 | 13.3 | 12 | 6.7 | 26 | 34.7 | 11 | 18.0 | 16 | 25.8 | 2 | 10.5% | 6 | 35.3 | 208 | 12.6% |
| BMI [n] * | 453 | | 404 | | 275 | | 176 | | 73 | | 60 | | 61 | | 18 | | 17 | | 1,626 | |
| Mean [kg/m^2^] ±SD [kg/m^2^] | 26.8 | ±5.3 | 26.8 | ±5.3 | 26.5 | ±5.2 | 26.4 | ±4.9 | 27.3 | ±5.2 | 27.2 | ±5.8 | 27.1 | ±5.1 | 27.4 | ±3.6 | 28.0 | ±5.4 | 26.8 | ±5.2 |
| Obese (BMI>30) [n, %] | 116 | 25.4 | 102 | 24.7 | 59 | 21.1 | 37 | 20.8 | 18 | 24.0 | 17 | 27.9 | 13 | 21.0 | 4 | 21.1 | 6 | 35.3 | 394 | 24.2 |
|  | **n** | **%** | **n** | **%** | **n** | **%** | **n** | **%** | **n** | **%** | **n** | **%** | **n** | **%** | **n** | **%** | **n** | **%** | **n** | **%** |
| Comorbidities [n] * | 456 |  | 413 |  | 279 |  | 178 |  | 75 |  | 61 |  | 62 |  | 19 |  | 17 |  | 1,650 |  |
| Any comorbidity | 235 | 51.5 | 192 | 46.5 | 153 | 54.8 | 91 | 51.1 | 52 | 69.3 | 35 | 57.4 | 43 | 69.4 | 13 | 68.4 | 12 | 70.6 | 876 | 53.1 |
| CCI =0 | 394 | 86.4 | 376 | 91.0 | 245 | 87.8 | 165 | 92.7 | 57 | 76.0 | 50 | 82.0 | 52 | 83.9 | 17 | 89.5 | 11 | 64.7 | 1,443 | 87.5 |
| CCI =1 | 29 | 6.4 | 13 | 3.1 | 15 | 5.4 | 4 | 2.2 | 2 | 2.7 | 1 | 1.6 | 2 | 3.2 | 2 | 10.5 | 2 | 11.8 | 71 | 4.3 |
| CCI ≥2 | 33 | 7.2 | 24 | 5.8 | 19 | 6.8 | 9 | 5 | 16 | 21.4 | 10 | 16.4 | 8 | 12.9 | - | - | 4 | 23.5 | 136 | 8.2 |
| Hypertension | 132 | 28.9 | 101 | 24.5 | 79 | 28.3 | 34 | 19.1 | 28 | 37.3 | 22 | 36.1 | 21 | 33.9 | 7 | 36.8 | 7 | 41.2 | 458 | 27.8 |
| Diabetes | 35 | 7.7 | 24 | 5.8 | 21 | 7.5 | 9 | 5.1 | 14 | 18.7 | 5 | 8.2 | 8 | 12.9 | 1 | 5.3 | 2 | 11.8 | 128 | 7.8 |
| Cardiovascular disorders | 6 | 1.3 | 3 | 0.7 | 5 | 1.8 | 1 | 0.6 | 10 | 13.3 | 5 | 8.2 | 2 | 3.2 | - | - | 3 | 17.6 | 39 | 2.4 |
| Tumour subtype [n] * | 442 |  | 403 |  | 275 |  | 175 |  | 73 |  | 61 |  | 61 |  | 18 |  | 16 |  | 1,611 |  |
| HR-positive/HER2-negative | 301 | 68.1 | 254 | 63.0 | 165 | 60.0 | 104 | 59.4 | 51 | 69.9 | 5 | 8.2 | 31 | 50.8 | 5 | 27.8 | 5 | 31.3 | 949 | 58.9 |
| HER2-positive | 83 | 18.8 | 87 | 21.6 | 61 | 22.2 | 29 | 16.6 | 8 | 11.0 | 51 | 83.6 | 26 | 42.6 | 10 | 55.6 | 7 | 43.8 | 402 | 25.0 |
| Triple negative | 58 | 13.1 | 62 | 15.4 | 49 | 17.8 | 42 | 24.0 | 14 | 19.2 | 5 | 8.2 | 4 | 6.6 | 3 | 16.7 | 4 | 25.0 | 260 | 16.1 |
|  | **F+A+C**  **n=456** | | **F+A+C+D n=413** | | **A+C+P**  **n=279** | | **A+C+D**  **n=178** | | **C+D**  **n=75** | | **Car+D**  **n=61** | | **A+C**  **n=62** | | **F+A+C+P n=19** | | **CMF**  **n=17** | | **Total**  **N=1,650** | |
|  | **n** | **%** | **n** | **%** | **n** | **%** | **n** | **%** | **n** | **%** | **n** | **%** | **n** | **%** | **n** | **%** | **n** | **%** | **n** | **%** |
| Tumour stage *^†^ | 403 |  | 364 |  | 248 |  | 162 |  | 63 |  | 51 |  | 54 |  | 16 |  | 15 |  | 1,456 |  |
| I | 197 | 48.9 | 59 | 16.2 | 33 | 13.3 | 31 | 19.1 | 20 | 31.7 | 16 | 31.4 | 19 | 35.2 | 5 | 31.3 | 11 | 73.3 | 407 | 28.0 |
| II | 194 | 48.1 | 245 | 67.3 | 115 | 46.4 | 80 | 49.4 | 31 | 49.2 | 23 | 45.1 | 25 | 46.3 | 10 | 62.5 | 2 | 13.3 | 763 | 52.4 |
| III | 12 | 3.0 | 60 | 16.5 | 100 | 40.3 | 51 | 31.5 | 12 | 19.0 | 12 | 23.5 | 10 | 18.5 | 1 | 6.3 | 2 | 13.3 | 286 | 19.6 |
| Nodal stage * | 456 |  | 413 |  | 279 |  | 178 |  | 75 |  | 61 |  | 62 |  | 19 |  | 17 |  | 1,650 |  |
| N- | 376 | 82.5 | 119 | 28.8 | 81 | 29.0 | 58 | 32.6 | 38 | 50.7 | 29 | 47.5 | 32 | 51.6 | 8 | 42.1 | 13 | 76.5 | 781 | 47.3 |
| N+ | 77 | 16.9 | 291 | 70.5 | 195 | 69.9 | 117 | 65.7 | 37 | 49.3 | 31 | 50.8 | 29 | 46.8 | 11 | 57.9 | 3 | 17.6 | 853 | 51.7 |
| NX | 3 | 0.7 | 3 | 0.7 | 3 | 1.1 | 3 | 1.7 | - | - | 1 | 1.6 | 1 | 1.6 | - | - | 1 | 5.9 | 16 | 1.0 |
| Menopausal status * | 456 |  | 412 |  | 278 |  | 178 |  | 75 |  | 61 |  | 62 |  | 19 |  | 17 |  | 1,648 |  |
| Premenopausal | 114 | 25.0 | 113 | 27.4 | 79 | 28.4 | 58 | 32.6 | 14 | 18.7 | 20 | 32.8 | 15 | 24.2 | 4 | 0.2 | 2 | 11.8 | 433 | 26.3 |
| Perimenopausal | 25 | 5.5 | 22 | 5.3 | 16 | 5.8 | 9 | 5.1 | - | - | 4 | 6.6 | - | - | 1 | 0.1 | - | - | 80 | 4.9 |
| Postmenopausal | 269 | 59.0 | 233 | 56.6 | 151 | 54.3 | 93 | 52.2 | 57 | 76.0 | 35 | 57.4 | 40 | 64.5 | 10 | 0.5 | 13 | 76.5 | 962 | 58.4 |
| Unknown | 48 | 10.5 | 44 | 10.7 | 32 | 11.5 | 18 | 10.1 | 4 | 5.3 | 2 | 3.3 | 7 | 11.3 | 4 | 0.2 | 2 | 11.8 | 173 | 10.5 |
| Local therapy * | 456 |  | 413 |  | 279 |  | 178 |  | 75 |  | 61 |  | 62 |  | 19 |  | 17 |  |  |  |
| BCS | 362 | 79.4 | 296 | 71.7 | 173 | 62.0 | 120 | 67.4 | 55 | 73.3 | 36 | 59.0 | 41 | 66.1 | 15 | 78.9 | 13 | 76.5 | 1,166 | 70.7 |
| Post-BCS radiotherapy  ^††^ | 322 | 89.0 | 258 | 87.2 | 138 | 79.8 | 109 | 90.8 | 49 | 89.1 | 29 | 80.6 | 29 | 70.7 | 14 | 93.3 | 11 | 84.6 | 1,001 | 85.8 |
| Mastectomy | 87 | 19.1 | 105 | 25.4 | 92 | 33.0 | 50 | 28.1 | 18 | 24.0 | 24 | 39.3 | 21 | 33.9 | 4 | 21.1 | 4 | 23.5 | 435 | 26.4 |
| Post-mastectomy radiotherapy ^†††^ | 28 | 32.2 | 74 | 70.5 | 65 | 70.7 | 33 | 66.0 | 10 | 55.6 | 15 | 62.5 | 11 | 52.4 | 1 | 25.0 | 4 | 100.0 | 259 | 59.5 |
| Surgery unknown | 7 | 1.5 | 12 | 2.9 | 14 | 5.0 | 8 | 4.5 | 2 | 2.7 | 1 | 1.6 | - | - | - | - | - | - | 49 | 3.0 |

Only regimen used in at least 1% of patients are shown; each regimen could be administered with or without additional HER2-inhibitor trastuzumab and / or additional endocrine therapy

* Number of patients with data available on the respective parameter at time of enrolment.

^†^ Tumour stage according to American Joint Committee on Cancer. 7th ed. New York, NY: Springer; 2010

^††^ Percentages refer to all patients who received BCS

**^†††^** Percentages refer to all patients who received mastectomy

**Abbreviations:** A, Epirubicin or Doxorubicin | BCS, Breast Conserving Surgery | BMI, Body Mass Index │ C, Cyclophosphamide | CCI, Charlson Comorbidity Index | D, Docetaxel | Car, Carboplatin | F, Fluorouracil | M, Methotrexate | P, Paclitaxel | SD, standard deviation | yrs, years.
